# IL-4/IL-13 Stimulated Macrophages Enhance Breast Cancer Invasion Via Rho-GTPase Regulation of Synergistic VEGF/CCL-18 Signaling

**DOI:** 10.3389/fonc.2019.00456

**Published:** 2019-05-31

**Authors:** Andrew C. Little, Pragathi Pathanjeli, Zhifen Wu, Liwei Bao, Laura E. Goo, Joel A. Yates, C. Ryan Oliver, Matthew B. Soellner, Sofia D. Merajver

**Affiliations:** Department of Internal Medicine, Hematology-Oncology, Rogel Cancer Center, University of Michigan, Ann Arbor, MI, United States

**Keywords:** Rho (Rho GTPase), metastasis, invasion, breast cancer, migration

## Abstract

Tumor associated macrophages (TAMs) are increasingly recognized as major contributors to the metastatic progression of breast cancer and enriched levels of TAMs often correlate with poor prognosis. Despite our current advances it remains unclear which subset of M2-like macrophages have the highest capacity to enhance the metastatic program and which mechanisms regulate this process. Effective targeting of macrophages that aid cancer progression requires knowledge of the specific mechanisms underlying their pro-metastatic actions, as to avoid the anticipated toxicities from generalized targeting of macrophages. To this end, we set out to understand the relationship between the regulation of tumor secretions by Rho-GTPases, which were previously demonstrated to affect them, macrophage differentiation, and the converse influence of macrophages on cancer cell phenotype. Our data show that IL-4/IL-13 *in vitro* differentiated M2a macrophages significantly increase migratory and invasive potential of breast cancer cells at a greater rate than M2b or M2c macrophages. Our previous work demonstrated that the Rho-GTPases are potent regulators of macrophage-induced migratory responses; therefore, we examined M2a-mediated responses in RhoA or RhoC knockout breast cancer cell models. We find that both RhoA and RhoC regulate migration and invasion in MDA-MB-231 and SUM-149 cells following stimulation with M2a conditioned media. Secretome analysis of M2a conditioned media reveals high levels of vascular endothelial growth factor (VEGF) and chemokine (C-C motif) ligand 18 (CCL-18). Results from our functional assays reveal that M2a TAMs synergistically utilize VEGF and CCL-18 to promote migratory and invasive responses. Lastly, we show that pretreatment with ROCK inhibitors Y-276332 or GSK42986A attenuated VEGF/CCL-18 and M2a-induced migration and invasion. These results support Rho-GTPase signaling regulates downstream responses induced by TAMs, offering a novel approach for the prevention of breast cancer metastasis by anti-RhoA/C therapies.

## Introduction

Since the mid-1990s, strategies to detect and treat early breast tumors have greatly improved, reflected in improved survival rates (1). However, breast cancer remains a serious disease, projected to claim the lives of nearly 42,000 women in in the US 2018 (1). Of the many factors that contribute to breast cancer-related mortality, metastatic spread is the most important. While some patients can live for years with late-stage metastasis, early diagnosis of metastatic dissemination offers no improvement to 5-year survival rates over diagnosing metastases when symptoms occur, most likely due to our current lack of available therapies specifically designed to target metastases and/or inhibit widespread cancer cell dissemination from a micrometastatic disease stage. Therefore, it is paramount that we enhance our understanding of the molecular mechanisms which drive the early stages of metastasis to enable realistic strategies to attenuate metastatic spread.

Cell migration is critical for normal development and physiology, although it can be aberrant in chronic inflammation and cancer metastasis. Over the last 15 years, it has been shown that signals from non-cancer cells in the tumor microenvironment (TME) contribute to enhancing the invasive phenotype of cancerous cells (2). Intriguingly, tumor-associated macrophages (TAMs) are the most abundant immune cell population in mammary tumors and are associated in multivariate analyses with elevated proportions of invasive tumor cells, high vascular grade, and reduced overall survival, pinning them as an independent biomarker of cancer severity and a prognostic indicator of metastatic progression (3–5). Macrophages are an inherently plastic cell population, readily switching between pro- (M1) and anti-inflammatory (M2) phenotypes depending on their environment (6, 7). In cancer, an environment of chronic inflammation is presumed to direct macrophage polarization toward an anti-inflammatory, M2-like phenotype (7). Under normal circumstances, the physiological role of M2 macrophages is to diminish inflammation to aid in tissue and/or epithelial wound repair (8). However, the features acquired by M2 macrophages in the TME have effects that are paradoxically associated with tumor progression; for instance, they facilitate and/or enable angiogenic responses, promote tumor growth, and eventually lead to tumor metastasis. Despite the rapidly growing number of studies which have characterized TAMs, the vast number of secreted factors by cancer cells and other cells of the TME leads to a diverse and transient TAM population that can readily switch between polarization states. Thus, characterizing which M2 population of macrophages (e.g., M2a, M2b, M2c) are specifically responsible for promoting tumorigenic outcomes in breast cancer remains an important, but not yet achieved goal.

The Rho family of GTPases are recognized for their role in directing cell migration. Aberrant regulation of the Rho-GTPases has been identified as an important contributing factor in the acquisition of the metastatic phenotype (9–12). Starting with Rac1 and RhoA (13, 14), other Rho-GTPases, including RhoC (15, 16), have been described for their specific roles in cellular motility, invasion, metastases, and angiogenesis (12, 17, 18). Our previous work showed that RhoC regulates inflammatory breast cancer migratory responses to macrophage conditioned media (19). Therefore, we hypothesized that RhoC plays a regulatory role specifically in TAM-induced breast cancer cell migratory and invasive responses.

In this study, our data defines IL-4/IL-13 stimulated macrophages (M2a macrophages) as the strongest inducers of breast cancer cell migration and invasion. Intriguingly, we find that both the Rho-GTPases RhoA and RhoC regulate M2a-induced responses to varying degrees. Our analysis of the M2a macrophage secretome confirms high levels of CCL-18 (20, 21). Importantly, our results showed significantly higher levels VEGF in M2a conditioned media vs. their M2b or M2c macrophage counterparts. Intriguingly, we find that CCL-18 and VEGF synergistically promote breast cancer migration and invasion, and this response is diminished via treatment of cells with the Rho-associated kinase (ROCK) inhibitor(s) Y-27632 or GSK429286A, delineating a unique targetable regulatory role for the Rho-GTPases. Collectively, these findings suggest therapeutic targeting of the Rho-GTPases may offer a novel approach for the prevention of breast cancer metastases.

## Materials and Methods

### Cell Models

Triple negative breast cancer (TNBC) MDA-MB-231 (MDA-231) cells were acquired from ATCC and maintained in Gibco RPMI-1640, 10% FBS, 5 μg/mL gentamycin, 2 mM L-glutamine, and 1X anti-anti. TNBC inflammatory cell model SUM-149 was kindly provided by Dr. Steve Ethier. SUM-149 cells were maintained in Gibco Ham's F12, 5% FBS, 0.5% penicillin-streptomycin, 2.5 μg/mL fungizone, 5 μg/mL gentamycin, 5 μg/mL insulin, 1 μg/mL hydrocortisone, and 2 mM L-glutamine. Randomized primary human female monocytes collected from whole blood were obtained from Astarte Biologics (Astarte, WA, USA). Human primary monocytes were cultured in X-VIVO 15 (Lonza, GA, USA), supplemented with 10% pooled human serum (Innovative Research, MI, USA), 50 ng/mL M-CSF, and 0.5% penicillin-streptomycin.

### Monocyte Polarization/Characterization, Macrophage Propagation, and Isolation of Conditioned Media (C.M.)

U937 monocytes were matured to macrophages with 50 ng/mL macrophage colony stimulating factor (M-CSF) for 24 h. Adherent macrophages were then polarized for 24 h with either 50 ng/mL IL-4 and IL-13 (M2a), ovalbumin-ovalbumin antibody immune complex extracts (IC; M2b), or 50 ng/mL IL-10 (M2c). IL-4, IL13, and IL-10 were purchased from R and D Systems (R&D, MN, USA). Following polarization, conditioned media was collected and concentrated with Amicon 3K MWCO spin columns to a final 10X concentration. Macrophage total RNA was collected and isolated using Qiagen RNeasy Mini kit (Qiagen, MD, USA). Total RNA was converted to cDNA using Promega AMV reverse transcriptase kit (Promega, WI, USA) and gene expression was evaluated by RT-qPCR using an ABI Quantstudio 3 (ABI, CA, USA). For RT-qPCR primers sequences and efficiencies, Supplemental Table 1. Human monocytes were differentiated to macrophages in the presence of 50 ng/mL M-CSF for 10–12 days until fully adherent and displayed proper macrophage morphology and size. Macrophages were then challenged with either 50 ng/mL IL-4 and IL-13 or vehicle control, daily for 4 days. Conditioned media was collected daily for 4 days and frozen at −80°C until concentration. Human macrophage conditioned media was concentrated in the exact fashion as described above for further use.

### Migration and Invasion Assays

For 2D migration, we employed a wound closure assay using Ibidi wound closure inserts. Cells were seeded at 7 × 10^5^ per mL and allowed to adhere overnight e.g., ~16 h. Following insert removal, cells were supplemented with various treatments, and wound closure was imaged. For 3D invasion, cells were seeded at 3 × 10^4^ in Corning ultra-low attachment spheroid round bottom 96-well plates and allowed to form spheroids for 3 days. Following spheroid formation, cells were embedded in an invasion matrix (Trevigen, CT, USA) and invasion was monitored over 6 days. For donut migration, technical details can be found here (22). For transwell migration assays, cells were seeded at 1 × 10^5^ in the apical chamber of Corning BioCoat Matrigel Invasion Chambers (Corning #354480, USA) and allowed to invade for 24 h. Invaded cells were counted manually. All images were acquired on the BioTek Cytation 5 imaging station, in tandem with the BioTek BioSpa automated incubator and robotics system (BioTek, VT, USA). For each of the cell treatments, C.M. was supplemented at a final 1X concentration; 20 ng/mL VEGF, CCL-18, or IL-4; 1 μM Y-27632 or GSK429286A (Tocris, R&D, USA).

### ELISA; Secreted Protein Evaluation, Cytokine Removal Assays

Aliquots of conditioned media isolates were submitted to the University of Michigan Immunology Core for pre-validated ELISA assays. Raw ELISA data was corrected for whole protein content determined by BCA (Pierce, Thermo Fisher). Corrected ELISA data was averaged across 4 independent experiments and plotted in a heat map using the free online matrix visualization software Morpheus (Broad Institute, USA). Statistical significance was determined by one-way ANOVA. For cytokine removal assays, we treated 10X M2a conditioned media with 10 μg/mL VEGF (R&D Systems #MAB293-100), CCL-18 (R&D #AF394), or a combination of VEGF and CCL-18 antibodies. Non-specific IgG_2B_ or IgG antibodies (10 μg/mL) were used as controls for VEGF or CCL-18, respectively.

### Cell Proliferation

Cells were seeded in 96 well formats and supplemented with various treatment regiments. Cell proliferation was monitored every 12 h using Promega ATP Cell Titer Glo reagent, per manufacturers protocol (Promega, WI, USA). For label free cell growth, we utilized image-based cellular identification strategies on our BioTek BioSpa-Cytation 5 automated high throughput imaging station. Cell size thresholds and background removal were applied for either MDA-231 or SUM-149 cells, and cells were counted over the course of 4 days. Statistical significance was determined by one-way ANOVA.

## Results

### IL-4/IL-13 Polarized Macrophages Are the Strongest Enhancers of Breast Cancer Cell Migration and Invasion

**Figure 1:**
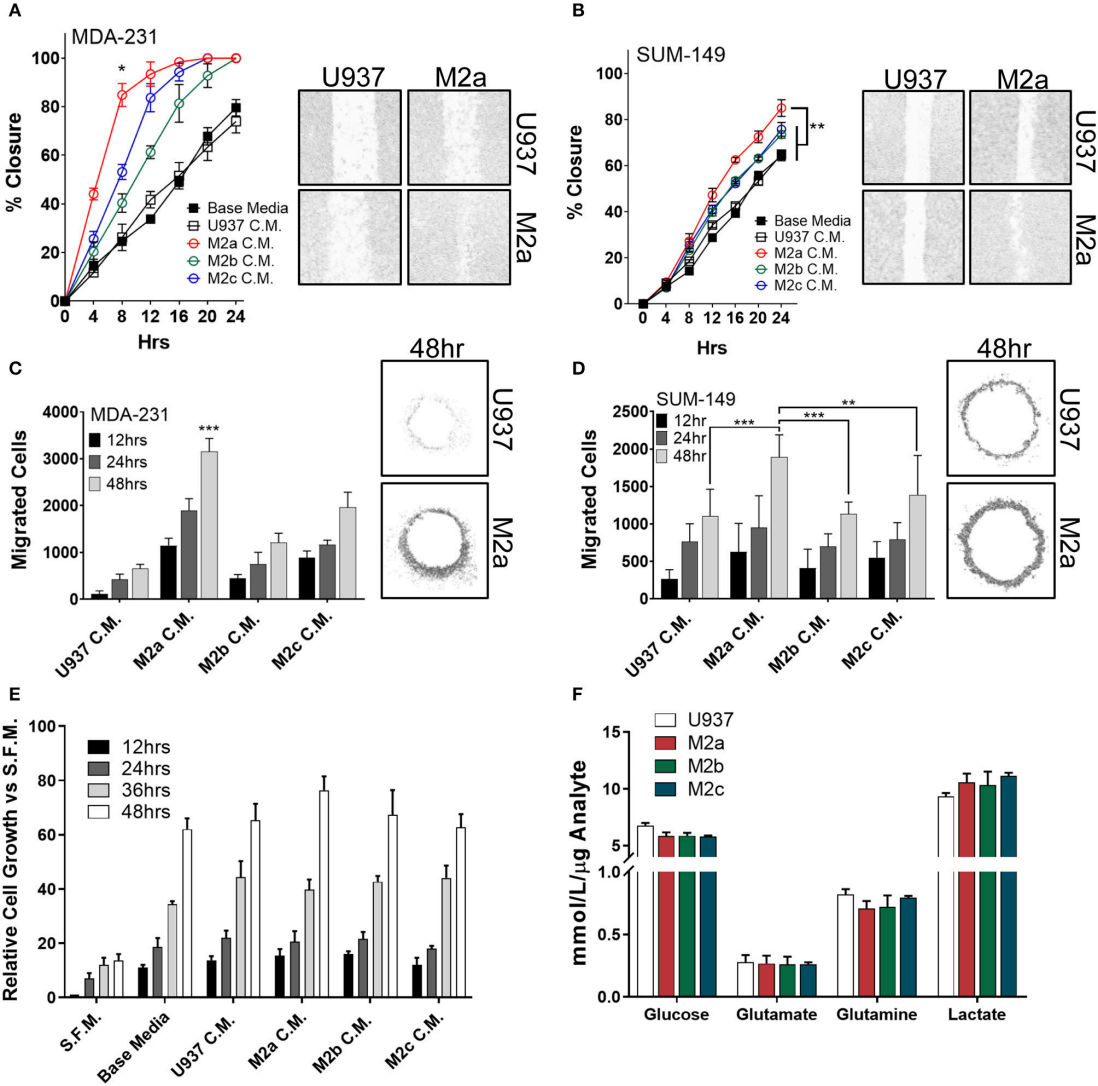
M2a macrophage conditioned media is a potent inducer of breast cancer cell 2D migration. Results from scratch wound migration assays display M2a conditioned media elicits a significantly greater migratory response in MDA-231 cells **(A)** and SUM-149 cells **(B)**. Similar results observed in a modified donut cell migration assay in MDA-231 **(C)** and SUM-149 cells (**D)**; 48 h post M2a C.M. addition in MDA-231 cells is statistically significant to all other 48 h time points, *p* < 0.001 **(C)**. The three M2 macrophage conditioned medias do not alter cellular growth rates in MDA-231 cells **(E)**. Results from YSI metabolite analysis display no changes in key metabolic analytes among the three M2 macrophages **(F)**. All results are compiled from ≥2 independent experiments; error bars represent ± standard deviation; Statistical significance determined by one-way ANOVA; *p*-value is displayed as *p < 0.05, **p < 0.01, ***p < 0.001.

To examine the effects the diverse M2-like macrophage populations had on breast cancer cells, we first induced *in vitro* monocyte-to-macrophage polarization by the addition of M-CSF to U937 monocyte cells. Following macrophage differentiation and cell adhesion, we stimulated macrophages with either recombinant IL-4/IL-13 (to promote an M2a phenotype), ovalbumin-ovalbumin antibody conjugate (to promote the M2b phenotype), or recombinant IL-10 (to promote an M2c phenotype) (Supplementary Figures S1A,B). To confirm polarization, we surveyed each population's RNA expression profile using reverse transcriptase-quantitative PCR (RT-qPCR) (Supplementary Figure S1C). Primer efficiency for RT-qPCR primers utilized in this study were verified to ensure fidelity, and primer sequences are listed in Supplementary Table 1. To study the effects of the three M2-like macrophages on breast cancer cell motility, we collected conditioned media from the three populations, concentrated them 10-fold, and supplemented cancer cells with a 1X final dilution of TAM-conditioned media. Stimulation with each of the three M2-macrophage conditioned media enhanced migration in wound closure assays in the TNBC MDA-MB-231 cell model (MDA-231) (Figure 1A), as well as the inflammatory TNBC cell line SUM-149 (Figure 1B). Specifically, stimulation with conditioned media from M2a macrophages enhanced both MDA-231 and SUM-149 cell migration in wound closure assays greater than conditioned media from either M2b or M2c macrophages (Figures 1A,B).

To independently test our results from wound closure assays, we employed a modified donut assay to assess 2D migration (22, 23). Results from the donut assay support our findings from wound closure assays, whereas stimulation of MDA-231 cells (Figure 1C) or SUM-149 cells (Figure 1D) with macrophage conditioned media produces an enhanced migratory response to M2a macrophages. To confirm that we were observing migration and not just increased proliferation, we stimulated cells in the same fashion as described in the migration assays and surveyed cell viability with ATP Cell Titer Glo reagent. No significant changes in cell numbers or proliferation were observed upon comparing stimulation with conditioned media from the three M2 macrophage populations (Figure 1E).

As macrophages and TAMs alike are known to alter their metabolism depending on their microenvironment and functional phenotypic requirements (24, 25), next we explored their metabolic adaptations in response to cancer cells. Metabolic flux of innate immune cells in the TME, such as altered levels of secreted metabolites (e.g., lactate), has been shown to influence cancer cell behavior (26, 27). This is of particular interest in inflammatory breast cancer, as recent findings from our lab show SUM-149 cells are heavily glycolytic, heavily dependent on glutamine for survival, and SUM-149 metabolism is regulated by RhoC (28). Therefore, we surveyed levels of consumed glucose, glutamate, and glutamine, while simultaneously examining secreted levels of lactate in the cell culture medium of polarized M2a, M2b, or M2c macrophages. We did not observe any unique differences between the three M2 macrophages (Figure 1F), suggesting that their metabolic profiles are not significantly contributing to enhancing breast cancer cell migration.

**Figure 2:**
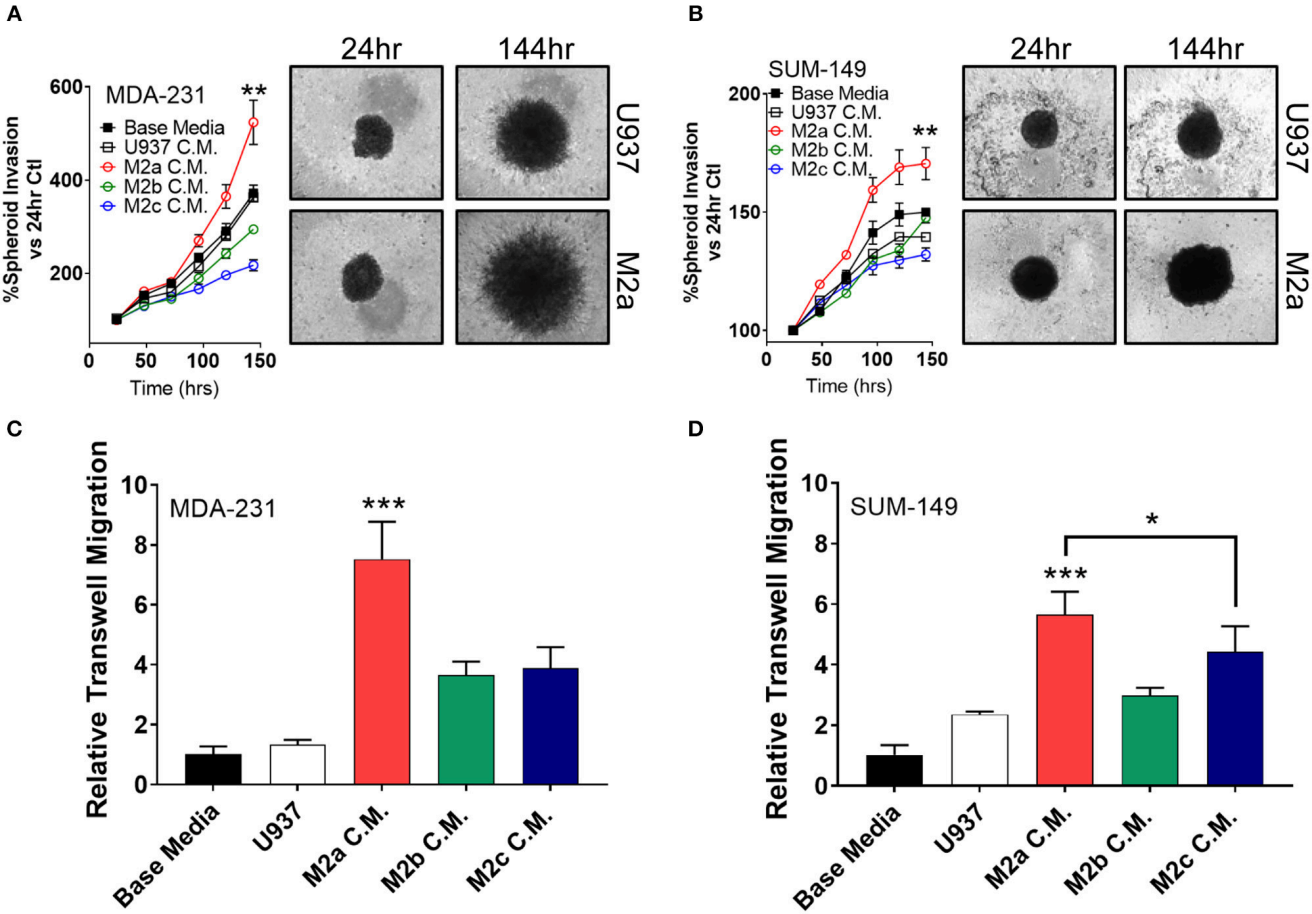
3D cell invasion assays confirm M2a TAMs as the greatest enhancer of breast cancer cell invasion. **(A)** Results from spheroid invasion assays in MDA-231 cells display a strong response to M2a conditioned media (C.M.). **(B)** Spheroid invasion results from the inflammatory TNBC cell line, SUM-149. Statistical significance in **(A,B)** is displayed as the 144 h time point compared against all other 144 h time points. **(C,D)** Results from 3D transwell migration confirm M2a conditioned media (red) enhances MDA-MB-231 **(C)** and SUM-149 **(D)** cancer cell invasion. All results are compiled from ≥2 independent experiments; Statistical significance was determined by one-way ANOVA; error bars represent ± standard deviation; *p*-value is displayed as *p < 0.05, **p < 0.01, ***p < 0.001.

Two-dimensional cell migration offers insight to unidirectional cellular motility, but it is a poor model for tumor cell invasion, as it does not recapitulate many of the key features of an *in vivo* tumor, such as extracellular matrix (ECM) components and 3D sphere-like growth. Therefore, we next aimed to test M2 macrophage-induced effects on tumor cell invasion specifically in 3D formats. To this end, we employed a spheroid invasion assay. MDA-231 cells or SUM-149 cells were embedded in Trevigen ECM matrix, supplemented with M2a, M2b, or M2c conditioned media, and allowed to propagate and invade for 6 days. Over this period, MDA-231 cells had a significantly greater invasive response to M2a conditioned media than controls (Figure 2A). Surprisingly, M2b or M2c conditioned media had a suppressive effect on MDA-231 spheroid invasion, contrasting the results we observed in 2D migration assays (Figure 2A). Similarly, M2b and M2c were suppressive for 3D invasion in the SUM-149 cells, although to a lesser extent (Figure 2B). This difference could be accounted by SUM-149's slower migratory rates than MDA-231 cells. To examine if M2a-induced responses were sufficient to induce migration in normal breast cells, we performed the spheroid invasion assay in human mammary epithelial cells and stimulated them with the various M2 conditioned media. Importantly, no significant differences were observed in hME cells (Supplementary Figure S2), supporting that the response to macrophages may be particular to transformed breast cancer cells.

As an orthogonal approach to the spheroid invasion/growth assay, we utilized a transwell migration assay where cells seeded in an apical transwell insert must traverse through a collagen layer to seed and colonize the basolateral side of the insert. Again, M2a conditioned media induced MDA-231 and SUM-149 cell invasion at a significantly higher rate than M2b or M2c conditioned media (Figures 2C,D), further supporting our findings from the spheroid invasion experiments. These results show that M2a polarized macrophages strongly and specifically enhance breast cancer cell migration and invasion in 3D.

### Rho-GTPases RhoA and RhoC Regulate M2a Induced Migratory Responses in Breast Cancer Cells

**Figure 3:**
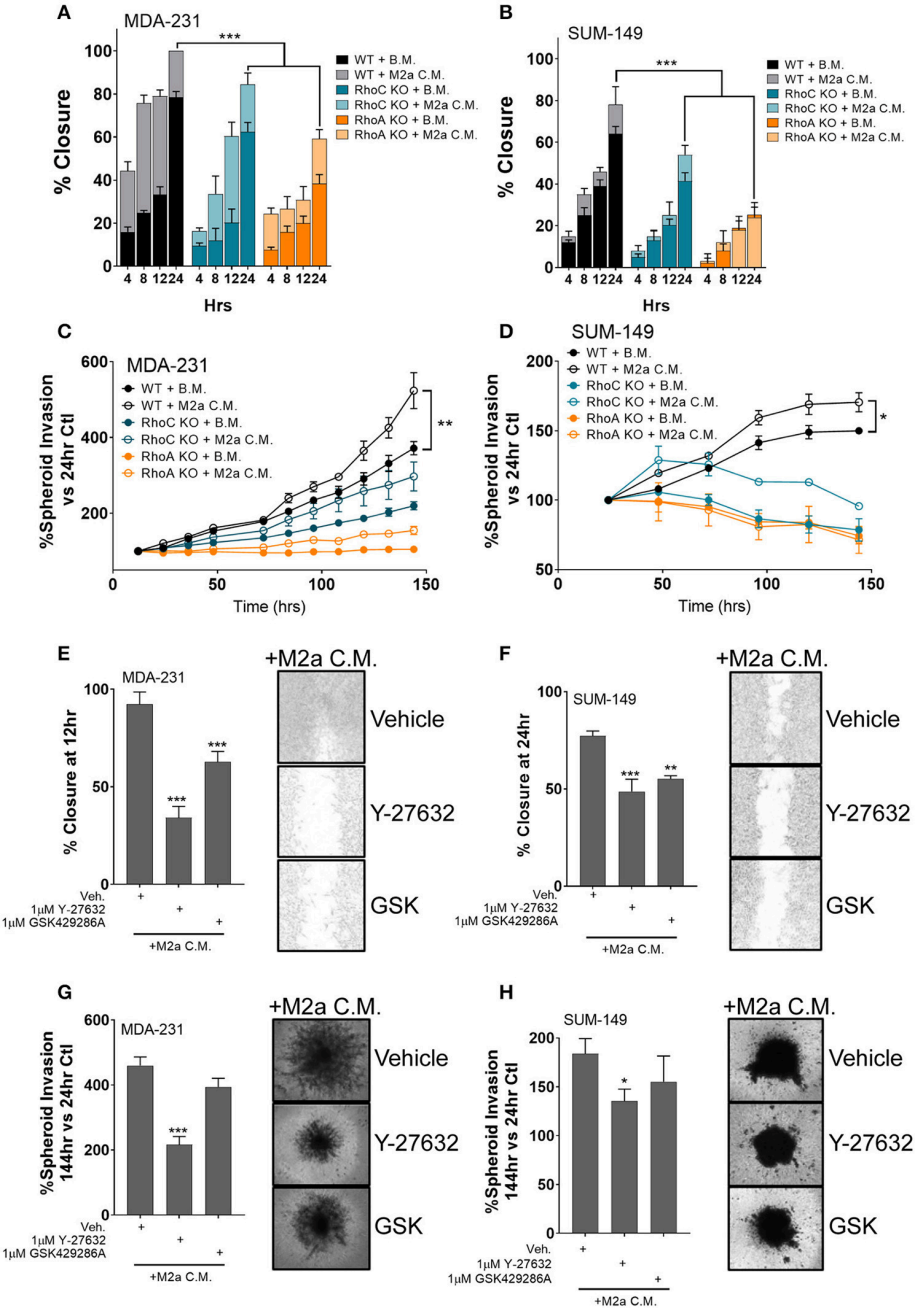
The Rho-GTPases regulate migratory responses to M2a macrophage conditioned media. Scratch assays results display a reduction in M2a-induced migration in both MDA-MB-231 **(A)** and SUM-149 cells **(B)**. Similar results obtained from 3D spheroid invasion assays **(C: MDA-231; D: SUM-149)**. Pretreatment with 1μM ROCK inhibitor (either Y-27632 or GSK429286A e.g. “GSK”) reduces M2a macrophage-induced 2D migration or 3D spheroid invasion in both MDA-231 **(E,G)** and SUM-149 cells **(F,H)**. All results are compiled from ≥2 independent experiments; error bars represent ± standard deviation; *p*-value is displayed as *p < 0.05, **p < 0.01, ***p < 0.001.

While an extensive body of research has characterized the Rho-GTPases role in the regulation of cancer cell motility, here we aim to understand the direct impact the Rho-GTPases have on communication signals from the tumor microenvironment to the cancer cell, a question that has remained largely unexplored. While RhoC is imperative for developmental processes (29–31), RhoC is also a crucial regulator of metastatic progression in various cancers likely due to its dysregulation (11, 32, 33). Thus, a detailed understanding of how it regulates the cancer cell responses to pro-tumorigenic macrophages would open new therapeutic strategies. Our previous report proved that the Rho-GTPase RhoC was necessary to regulate macrophage induced migration of the inflammatory breast cancer cell model SUM-149 (19). Therefore, we hypothesized that RhoC, and potentially RhoA, may both regulate TAM-induced migratory responses, particularly in response to M2a TAMs. To test the role of RhoA and RhoC in M2a-induced breast cancer cell migration, we stimulated MDA-231 or SUM-149 wild type (WT), RhoA CRISPR-Cas9 knockout (ΔRhoA), or RhoC CRISPR-Cas9 knockout (ΔRhoC) with either base media or M2a conditioned media and monitored wound closure over 24 h. Interestingly, both RhoA and RhoC knockout had significant and major impact on M2a-induced migration in both the MDA-231 cells (Figure 3A) and the SUM-149 cells (Figure 3B).

While both RhoA and RhoC appear to regulate M2a-induced responses, our data supports that RhoA is a quantitatively more critical mediator of TAM induced migration, as we observe significantly diminished migration in the RhoA knockout cell lines (Figures 3A,B). These results are supported in a 3D spheroid invasion assay, as the RhoC and RhoA knockout lines do not display a significant response to M2a-conditioned media in either MDA-231 (Figure 3C) or SUM-149 cells (Figure 3D). These data confirm that the Rho-GTPases, RhoA and RhoC, both regulate M2a-induced metastatic responses. Despite the overall diminished migratory/invasive responses in our RhoA or RhoC knockout cell lines, conditioned media from M2a macrophages is still able to elicit a pro-migratory effect. These results strongly support that RhoA and RhoC harbor unique and independent regulatory roles in breast cancer migration.

Stimulation of the Rho-GTPases activates their downstream kinase, Rho-associated protein kinase (ROCK), known to regulate cell motility (17). Our data suggest that RhoA and RhoC both harbor regulatory roles in mediating M2a macrophage induced responses; therefore, we hypothesized that responses may be directed through ROCK signaling. To test this, we pretreated MDA-231 cells or SUM-149 cells with the ROCK inhibitors Y-27632 or GSK429286A (GSK) and surveyed migratory responses to M2a conditioned media in a 2D migration assay. Indeed, ROCK inhibition slowed M2a-induced migration rates in both MDA-231 (Figure 3E) and SUM-149 cells (Figure 3F). We next aimed to determine if ROCK signaling regulated 3D invasion. We observe similar results in the 3D spheroid invasion assay in both the MDA-231 (Figure 3G) and SUM-149 cells (Figure 3H). Collectively, these findings show that Rho-GTPases regulate M2a-induced migratory and invasive responses in both MDA-231 and SUM-149 cells.

### M2a Macrophage Derived CCL-18 and VEGF Synergistically Enhance the Breast Cancer Metastatic Phenotype, Regulated by ROCK Signaling

**Figure 4:**
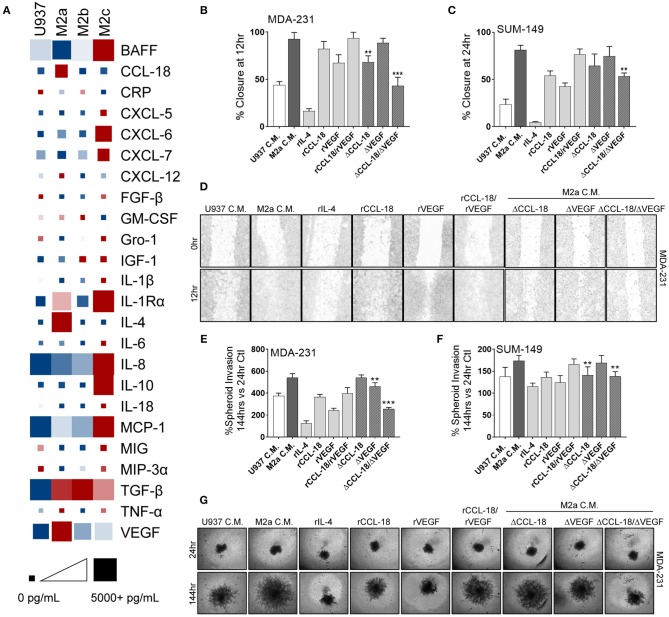
Synergistic activity of CCL-18 and VEGF, secreted from M2a macrophages, enhance breast cancer cell migration. **(A)** ELISA results from concentrated cell supernatants indicate that M2a macrophages secrete higher levels of CCL-18, IL-4, and VEGF than their M2b or M2c counterparts. ELISA data is averaged across 4 independent experiments. Colors indicate row comparisons of a single analyte protein level [low levels (blue) vs. high levels (red)] compared among the macrophage populations. Square or box size is designed to provide quantitative global analysis of overall analyte protein levels e g., 1 pg/mL (small square) to 5,000 pg/mL (large square). Results from wound closure assay in MDA-MB-231 cells **(B)** and in SUM-149 cells **(C)** show removal of CCL-18 and VEGF from M2a C.M. significantly inhibits migration. **(D)** Representative images of MDA-MB-231 cells wound migration assays results. Similar results obtained from spheroid invasion assays in both MDA-231 **(E)** and SUM-149 cells **(F)**. Representative images from MDA-231 invasion assays shown in **(G)**. Data is reported as an average ±std. dev. of 3 independent experiments, analyzed by one-way ANOVA, **p > 0.01, ***p > 0.001.

TAMs routinely secrete a large suite of cyto/chemokines, growth factors, and other components which can promote cancer cell extravasation, suppress innate immune function in the TME, and recruit other immune modulators to support cancer metastases (5, 34) by shielding cancer cells from immune mediated destruction. Therefore, it is critical to understand the mechanisms by which M2a TAMs promote breast cancer cell migration and invasion and how the Rho-GTPases regulate these processes. We surveyed conditioned media from U937 monocytes and the three M2-like macrophages for a large array of their potential secreted components. As others have reported (20, 21), we found significantly elevated levels of CCL-18 in M2a conditioned media vs. their other M2 macrophage counterparts (Figure 4A; Supplementary Figure S3). In parallel, we found significantly higher levels of IL-4 (data corrected to remove exogenously supplemented recombinant IL-4) and VEGF in M2a conditioned media (Figure 4A; Supplementary Figure S3). In contrast, we found significantly lower levels of chemokine (C-X-C motif) ligand 9 (CXCL-9) and chemokine (C-C motif) ligand 20 (CCL-20) in M2a conditioned media compared to the other M2 populations. We further aimed at understanding which of components were most salient in their influence on motility and invasion.

To evaluate which of these components were critically important in supporting breast cancer migration, we treated MDA-231 or SUM-149 cells with each of the individual proteins/cytokines and examined migratory responses. Our data exhibit strong migratory responses to recombinant CCL-18 (rCCL-18; 20 ng/mL) in both the MDA-231 and SUM-149 cells (Figures 4B–D). Exogenous addition of recombinant VEGF (rVEGF_165_; 20 ng/mL) promoted cell migration, although to a lesser extent than rCCL-18. Interestingly, stimulation with both VEGF and CCL-18 promoted the fastest rates of migration, suggesting synergistic, or complementary mechanisms of action. Surprisingly, treatment with recombinant IL-4 (rIL-4; 20 ng/mL) had no effect on cell migration (Figures 4B–D). As mentioned previously, ELISA analysis of M2a macrophage conditioned cell medium revealed lower levels of CCL-20 and CXCL-9 vs. M2b or M2c conditioned medias. Therefore, we tested whether CCL-20 or CXCL-9 had an inhibitory effect on M2a-induced migratory responses, as some contradiction in the literature exists as to the role of each of these cytokines (35, 36). Stimulation of either MDA-231 cells or SUM-149 cells with recombinant CXCL9 (rCXCL9; 50 ng/mL), recombinant CCL-20 (rCCL-20; 50 ng/mL), or a combination of both cytokines each had no inhibitory effect on M2a-mediated migration in a wound closure assay (Supplementary Figure S4). Again, we employed the 3D spheroid invasion assay to examine the potential invasive effects of CCL-18, VEGF, and IL-4. Indeed, we find that rVEGF and rCCL-18 are sufficient to promote an invasive phenotype and stimulation with the combination of rVEGF/rCCL-18 had the strongest invasive response in both the MDA-231 and SUM-149 cells (Figures 4E–G). Addition of rIL-4 had no impact on spheroid growth or invasion in either cell model.

Based on the finding that recombinant CCL-18 and VEGF cause a strong migratory and invasive response in our aggressive breast cancer cells, we removed each of these components from M2a conditioned media and supplemented cancer cells with either VEGF-depleted media (ΔVEGF), CCL-18-depleted media (ΔCCL-18), or both VEGF- and CCL-18-depleted conditioned media (ΔCCL-18/ΔVEGF). We confirmed removal of VEGF and/or CCL-18 in our M2a conditioned media by ELISA (Supplementary Figure S5). Our data show that CCL-18-depleted M2a conditioned media slowed migratory responses in 2D migration in both MDA-231 and SUM-149 cells, with no observed differences following VEGF removal (Figures 4B–D). Intriguingly, we see significantly slower rates of migration following removal of both CCL-18 and VEGF from M2a conditioned media (Figures 4B–D). CCL-18 or VEGF depletion from M2a media had varying effects for spheroid invasion, although our results consistently show diminished invasion following removal of both proteins from the M2a media (Figures 4E–G). Taken together these data strongly support that synergistic influence of VEGF and CCL-18 are critical for migratory/invasive responses of cancer cells to M2a macrophages.

**Figure 5:**
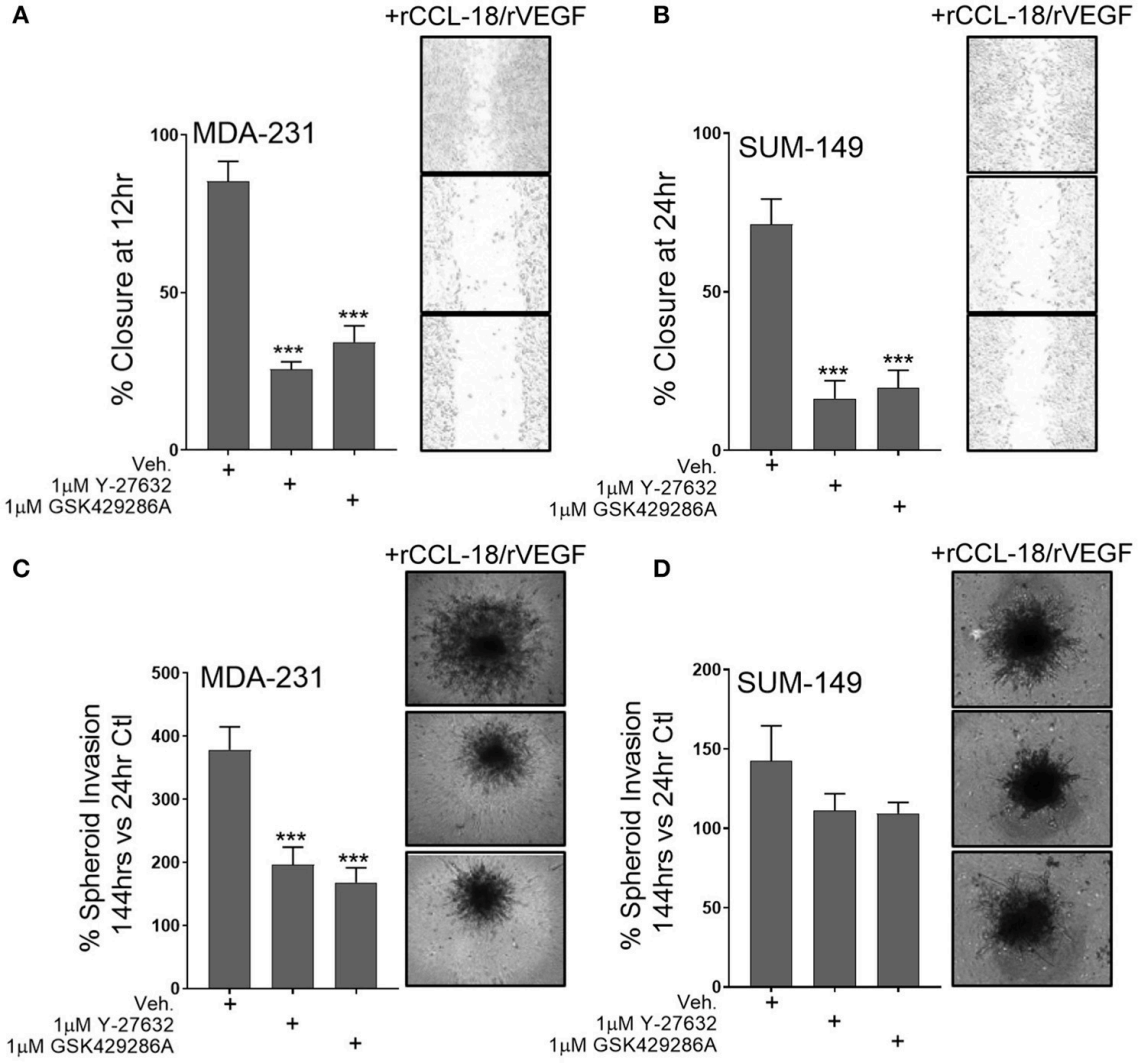
ROCK inhibition significantly diminishes synergistic VEGF/CCL-18 included breast cancer cell migration and invasion. **(A)** Wound closure rates are dramatically slowed in MDA-231 cells pretreated with either 1 μM Y-27632 or 1 μM GSK429286A followed by VEGF and CCL-18 administration. **(B)** Wound closure rates in SUM-149 cells are slowed following pretreatment with the ROCK inhibitors, Y-27632 or GSK429286A followed by rVEGF/CCL-18 administration. **(C)** Results from spheroid invasion assays display VEGF/CCL-18-mediated invasion is reduced in MDA-231 cells pretreated with either ROCK inhibitor. **(D)** ROCK inhibition in SUM-149 cells does not significantly inhibit cellular invasion. Data is reported as an average ±std. dev. of at least 3 independent experiments, analyzed by one-way ANOVA, ***p > 0.001.

Since our data shows that Rho-GTPase activation and downstream ROCK signaling regulates M2a-induced migration, we sought to understand whether ROCK signaling is also downstream of VEGF and/or CCL-18. To test this, we pretreated MDA-231 or SUM-149 cells with the ROCK inhibitors Y-27632 or GSK429286A and examined wound closure rates in the presence of rCCL-18 and rVEGF. As hypothesized, we find that pretreatment with the ROCK inhibitors significantly repress rCCL-18/rVEGF induced migratory responses in both MDA-231 and SUM-149 cells (Figures 5A,B). This result is recapitulated in MDA-231 invasion assays (Figure 5C) and a strong, but non-significant trend was observed in SUM-149 cells (Figure 5D). Again, to confirm migratory/invasive responses were not due to enhanced proliferation, we utilized label-free cell growth imaging assays. In parallel with our findings in Figure 1E, we did not observe any changes in proliferation following treatment with rVEGF, rCCL-18, or rVEGF and rCCL-18 combined (data not shown). Collectively, these data confirm that the Rho-GTPases and downstream ROCK signaling regulate CCL-18 and/or VEGF induced migratory responses.

### Primary Human M2a Macrophages Enhance Breast Cancer Cell Migration and Invasion Through ROCK Signaling

**Figure 6:**
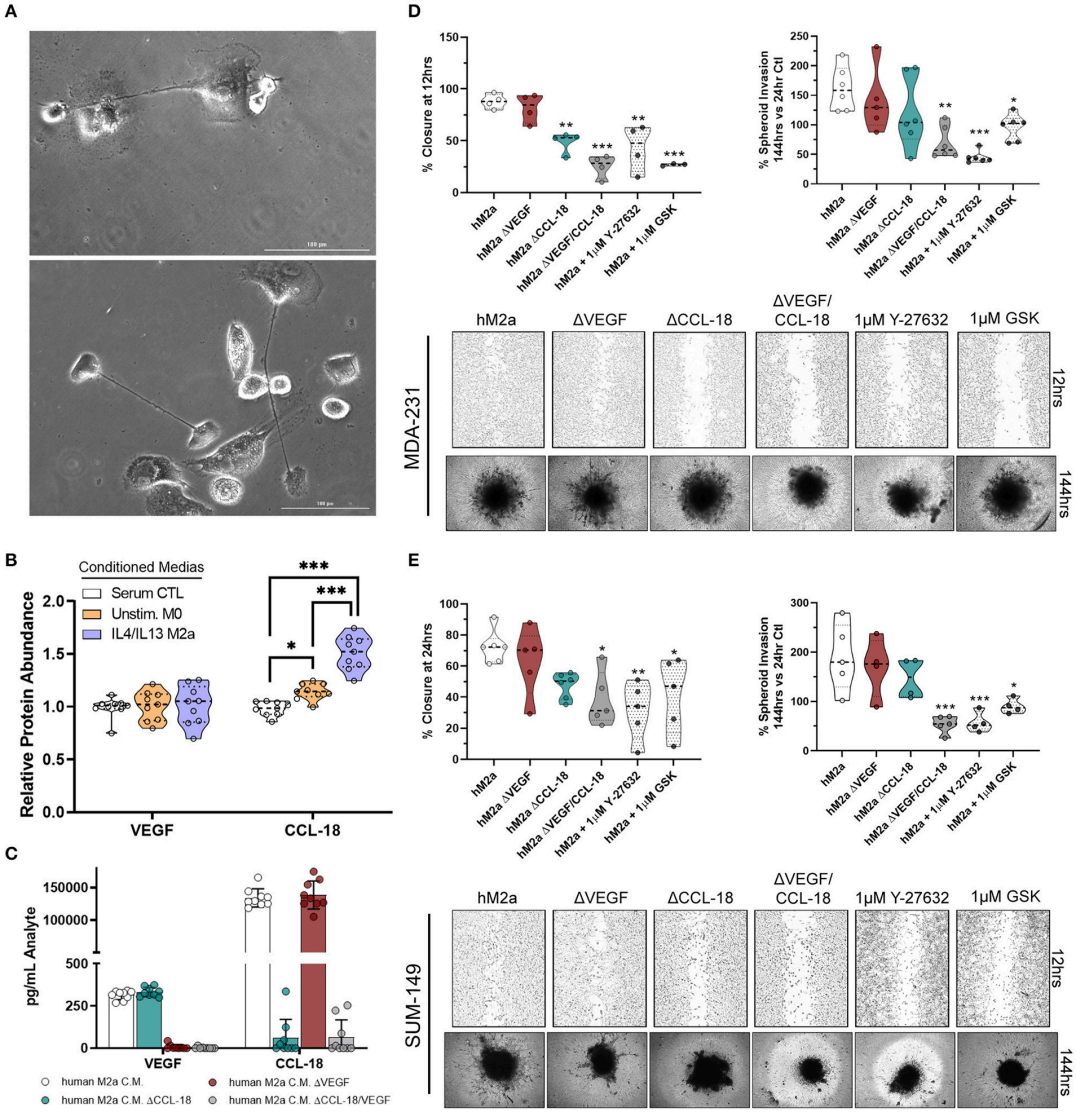
Human M2a macrophages enhance breast cancer cell migration and invasion through VEGF/CCL-18 signaling regulated by Rho-GTPases. 40X phase contrast images of fully differentiated human macrophages **(A)**. ELISA results from concentrated conditioned media extracts from normal human macrophage growth media (serum containing; white), unstimulated M-CSF differentiated macrophages (orange), and IL-4/IL-13 polarized human M2a macrophages (purple/blue) **(B)**. ELISA results from analyte removal assays **(C)**. Results of wound closure 12 h post wound (left panel) or spheroid invasion 144 h post treatment/supplementation (right panel) experiments utilizing either human M2a conditioned media (M2a) analyte depleted hM2a media (e.g., hM2a ΔVEGF) or pretreatment with ROCK inhibitors with hM2a macrophage conditioned media in MDA-231 cells **(D)**. Representative images of either wound closure (top) or spheroid invasion (bottom) experiments in MDA-231 cells are shown below quantified data. Similar results displayed for wound closure (left panel) or spheroid invasion (right panel) with representative images below quantified data for SUM-149 cells **(E)**. All results are compiled from ≥2 independent experiments; Statistical significance was determined by one-way ANOVA; error bars represent ± standard deviation; *p*-value is displayed as *p < 0.05, **p < 0.01, ***p < 0.001.

It is well-recognized that results obtained from cultured cell models can widely vary from primary cells and may not entirely recapitulate responses observed *in vivo*. This is of particular concern in macrophage biology as they are an inherently plastic cell population, readily changing polarization, genotype, and phenotype, depending on their environment. Therefore, we aimed to determine if M2a macrophages derived from primary human monocytes displayed similar features and impact breast cancer cell phenotypes as the U937-derived M2a macrophages. Using primary human monocytes derived from whole blood, we initially differentiated them into unstimulated human macrophages by the addition of 20 ng/mL M-CSF to human monocyte culture medium (Figure 6A). Over the course of 4 days, we stimulated with IL-4/IL-13, collected and concentrated the conditioned media daily, as described previously. Initially, we surveyed the levels of CCL-18 and VEGF produced in human M2a (hM2a) macrophages and compared them to unstimulated human primary macrophages. We observed no change in VEGF production from hM2a human primary macrophages (Figure 6B), which was overall very low both in the primary and in the differentiated subpopulation. In contrast, we observed significantly enhanced levels of CCL-18 secreted by hM2a macrophages as compared to unstimulated human macrophages (Figure 6B). We predicted that hM2a macrophages would secrete VEGF and CCL-18 at differing concentrations as compared to U937-derived M2a macrophages; indeed, we observed different absolute levels of both VEGF and CCL-18 in the two complementary models (Supplementary Figure 6; Supplementary Figure 3), although the response trend for CCL-18 was equivalent in both models. These data highlight the significant heterogeneity that may be present between primary cells and cell culture models, further imparting the importance of experimental replication in various models to validate critical results. Despite the observed differences in secreted VEGF/CCL-18 levels, we still asked whether hM2a macrophages influence breast cancer cell migration and invasion through synergistic VEGF/CCL-18 signaling, and if this process proceeds through ROCK signaling. We generated VEGF depleted (ΔVEGF), CCL-18 depleted (ΔCCL-18), or VEGF and CCL-18 (ΔVEGF/CCL-18) depleted hM2a conditioned media (Figure 6C). Interestingly, removal of VEGF from hM2a media had little impact on either MDA-231 or SUM-149 wound closure rates or spheroid invasion (Figures 6D,E). Importantly however, removal of CCL-18 from hM2a conditioned media slowed 2D migration and 3D invasion (Figures 6D,E), but only significantly in MDA-231 wound closure (Figure 6D). Confirming our initial findings, removal of both VEGF and CCL-18 had significant impacts on MDA-231 2D migration and 3D invasion (Figure 6D), and in SUM-149 2D migration and 3D invasion (Figure 6E). Next, we examined if blocking Rho-GTPase signaling via ROCK inhibition would be sufficient to diminish hM2a macrophage-induced breast cancer cell migration and invasion. Both ROCK inhibitors, Y-27632 or GSK429286A, effectively diminished hM2a macrophage-induced metastatic phenotypes in both MDA-231 (Figure 6D) and SUM-149 (Figure 6E). These results suggest that targeting of the Rho/ROCK pathway is a promising and viable strategy for the potential management of metastatic progression, especially in patients who have elevated levels of M2a TAMs.

## Discussion

The roles of the Rho-GTPases in regulating cell migration and invasion were initially established roughly 20 years ago. These studies largely focused on the precise mechanisms by which Rho-GTPases harbor intrinsic regulatory roles as well as characterizing cofactors and surveying downstream effector responses. Recently, studies have begun to illustrate the complex regulation of the Rho-GTPases in response to stimulation or secreted signals from the cellular microenvironment. This is particularly relevant with the re-emergence of tumor immunology based anti-cancer strategies and the development of modern immunotherapies. Understanding how cancer cells respond to the multifaceted, dynamic signals that derive from infiltrated immune cells in the TME is an especially critical problem for aggressive breast cancers, such as triple negative and inflammatory phenotypes, for which there are at present no targeted therapies based on signaling (outside of PARP inhibitors for BRCA germline mutation carriers). Since macrophages are the most abundant population of immune cells that reside in the TME, we sought to better understand macrophage-breast cancer cell communication and how this process is regulated in triple negative and inflammatory breast cancer, both of which account for a disproportionate burden of morbidity and mortality from breast cancer.

In our previous work, we observed that conditioned media extracts from *in vitro*, unpolarized macrophages significantly enhance migration in inflammatory breast cancer cells (such as SUM-149 cells) and that this process is regulated by RhoC (19). Macrophages are constantly adapting due to the variety of signals they encounter in the TME; therefore, in this study we aimed to understand their specific roles at the molecular level and better define the class of macrophages that is the main culprit in eliciting breast cancer metastatic progression. Here, we find that IL-4/IL-13 polarized M2a macrophages enhance breast cancer cell migration and invasion at a greater rate than either M2b or M2c macrophages and are thus an important subpopulation to target therapeutically. Moreover, we find that the Rho-GTPases RhoA and RhoC regulate M2a macrophage-induced responses through the synergistic effects of VEGF and CCL-18 signaling combined, and these effects can be attenuated by ROCK inhibition Collectively, our data strongly supports that use of ROCK inhibitors may be an effective strategy to diminish tumor invasion.

In a 2011 hallmark paper by Chen et al. CCL-18 from TAMs was found to promote breast cancer metastasis through the novel CCL-18 receptor, PITPNM3 (20). Since then, CCL-18 has been characterized as responsible for cancer progression in various cancer types (21, 37–41). Here, we provide evidence for a novel mechanism of Rho-GTPases regulating CCL-18-induced breast cancer migration. While direct targeting of CCL-18 or its receptor PITPNM3 or CCR8 (42, 43) seems like an attractive route for targeted therapy, this may have significant systemic side effects for patients, as CCL-18 signaling is critical for normal innate immune responses (43–45). As an alternative, our work suggests directing therapies toward Rho-GTPase signaling, for instance via ROCK inhibition, to diminish CCL-18 induced responses. Currently, ROCK inhibitors have been limited in their use in clinical trials as concerns over their potential systemic side effects are severe due to off-target effects of the existing compounds (46). Collectively, this work supports the need for better, more specific ROCK inhibitors. Overall, ROCK inhibition may be an efficacious, tolerable route for the management of metastatic breast cancer or other aggressive cancers and is worthy of further study.

Angiogenic factors are key contributors to cancer metastasis. VEGF is known to be secreted from a variety of cell types within the TME (e.g., cancer cells, TAMs, stromal cells, among others). Thus, our observation of enhanced secreted levels of VEGF from M2a macrophages is consistent with prior literature (21, 47). However, our data show the novel finding of synergy between VEGF and CCL-18 signaling in their ability to enhance breast cancer motility and invasiveness. While VEGF has clearly defined roles for enhancing tumor cell migration via stimulation of angiogenesis, CCL-18 is a key factor in the chemotaxis of naïve T-cells and immune-suppression in the TME. However, these two signaling pathways do not have any previously described overlap or co-operativity regarding their regulation of breast cancer cell motility. Here we delineate that CCL-18 and VEGF enhance breast cancer migration and invasion, potentially as a pre-angiogenic step. These data confirm the importance of the CCL-18/VEGF axis in breast cancer metastasis, further supporting the need for future studies of their combined roles in priming the TME for angiogenesis and tumor progression. This work is potentially especially timely and relevant to efforts to enhance the efficacy of immune therapies in aggressive tumors such as triple negative and inflammatory breast cancer, where the performance of the latter has been modest.

In summary, this work shows that IL-4/IL-13 stimulated M2a macrophages are the most potent enhancers of breast cancer migratory and invasive phenotypes and thus the sub-population most likely to have anti-cancer effects if targeted as a single modality or in combination with anti-PD-1 or anti-PD-L1 antibodies, to help prime the TME for immune therapies. While *in vivo* polarized TAMs most likely experience a large diversity of cyto/chemokines and exist in a continuum of activation states, characterization of the various TAM polarization states and how they affect breast cancer cell behavior is critical in understanding the mechanisms which promote cancer outcomes. Synergistic utilization of CCL-18 and VEGF by M2a macrophages offers insight as to how these cells enhance metastatic outgrowth, most likely a precursor to angiogenesis, and supports their targeting for therapeutic intervention. Additionally, our results displaying that CCL-18 and VEGF signaling proceed through the Rho-GTPases, is a novel observation. This supports that combination targeting of the Rho-GTPases and M2 macrophage activation (e.g., CSF1R inhibition) would be an effective strategy to suppress breast cancer cell invasion for use for example in the adjuvant setting in high-risk lesions or in combination with other chemo or immune therapies, which elicit cancer cell death. Collectively, these data show that the Rho-GTPases are critical in the regulation of M2a macrophage-induced migratory and invasive responses in breast cancer cells and offers unique therapeutic opportunities for the suppression of breast cancer metastatic spread.

## Author Contributions

AL and SM conceived the study. AL and PP performed majority of experiments for the study. ZW, LB, JY, and LG assisted in preparing and/or performing experiments for the study. AL, PP, and SM analyzed data. AL, JY, CO, MS, and SM discussed analyzed results and contributed key intellectual input to the study. AL wrote the manuscript and prepared figures. AL, JY, MS, and SM assisted in drafting and editing the final manuscript.

### Conflict of Interest Statement

The authors declare that the research was conducted in the absence of any commercial or financial relationships that could be construed as a potential conflict of interest.
