# Supplementary material for: IL-4/IL-13 Stimulated Macrophages Enhance Breast Cancer Invasion Via Rho-GTPase Regulation of Synergistic VEGF/CCL-18 Signaling
Source: Front Oncol. 2019 May 31;9:456. doi: 10.3389/fonc.2019.00456 (PMC6554436; doi:10.3389/fonc.2019.00456)
Supplement: Supplementary file 1 [file Data_Sheet_1.pdf]

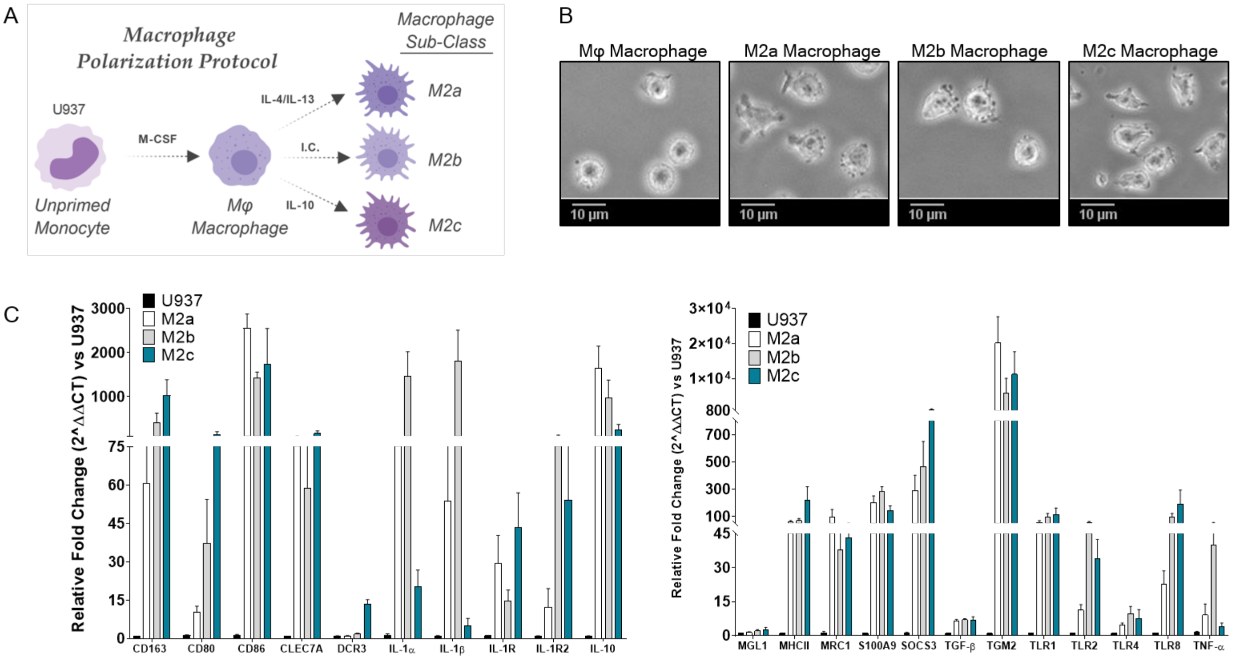

Supplemental Figure S1. **M2 macrophage polarization model and RT-qPCR characterization.** **(A)** Cartoon schematic depicting the *in vitro* polarization scheme for the M2 macrophage populations. **(B)** Phase images taken with a 20X objective shows the morphological features of the various M2 macrophage families. **(C)** qPCR results display expression profiles of the 3, *in vitro* polarized M2-like macrophages. Results of displayed as averages, cumulative of at least 3 independent experiments. Error bars represent standard deviation.

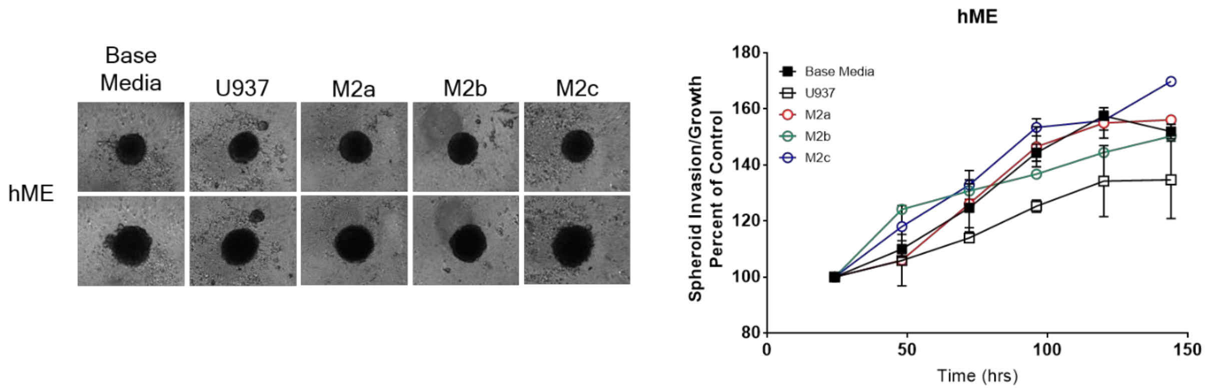

Supplemental Figure S2. Results from spheroid invasion assays display conditioned media from M2 macrophages has a minimal effect on human mammary epithelial cells (hME). Images are representative images selected from a single experiment (left). Averaged data is compiled from two independent experiments. Error bars represent standard deviation.

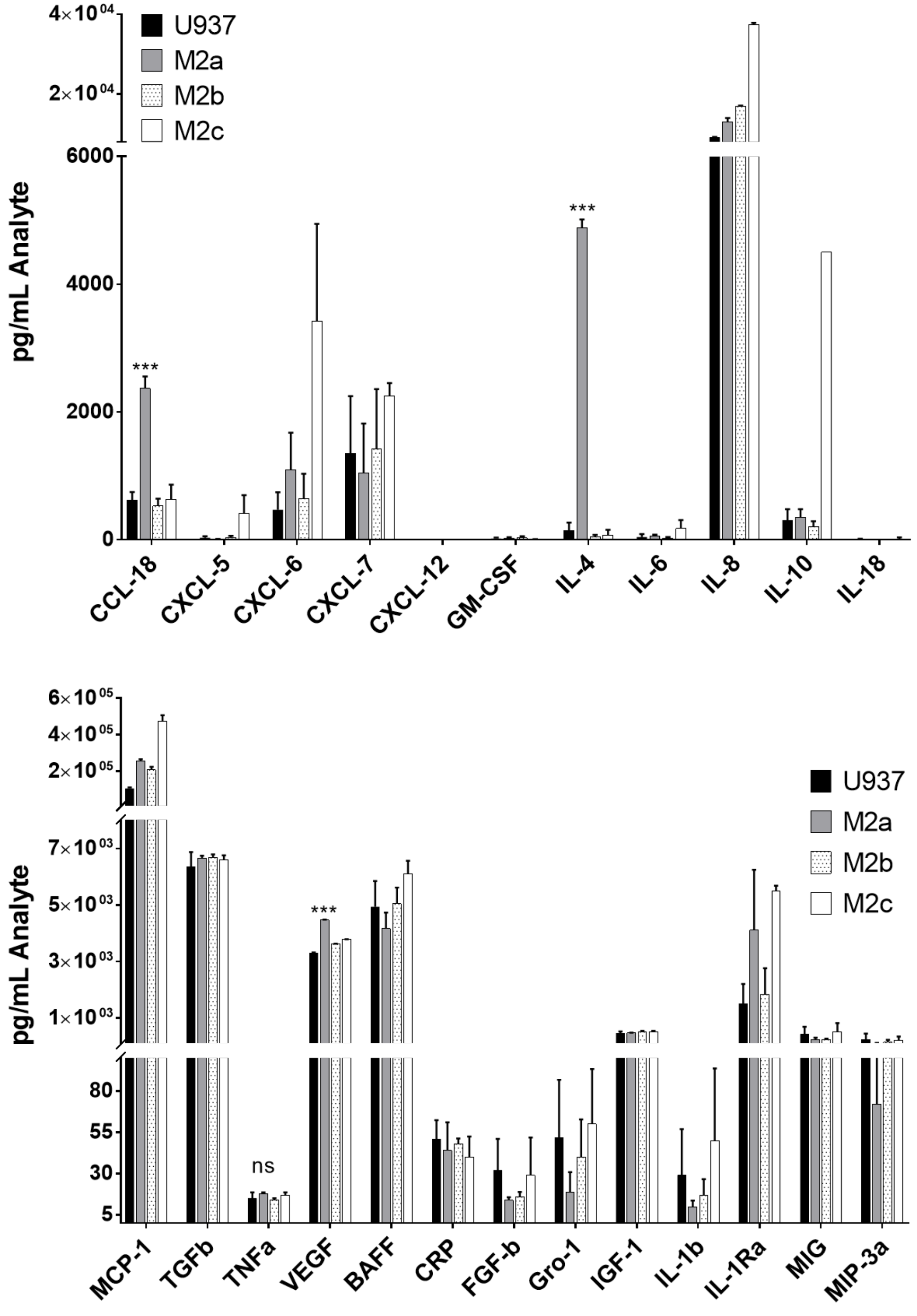

Supplemental Figure S3. ELISA results of various secreted components in the conditioned media isolated from either U937 monocyte or M2-macrophage cell populations. Averaged data is compiled from three independent experiments. Statistical significance was determined by one-way ANOVA; ***p<0.001. Error bars represent standard deviation.

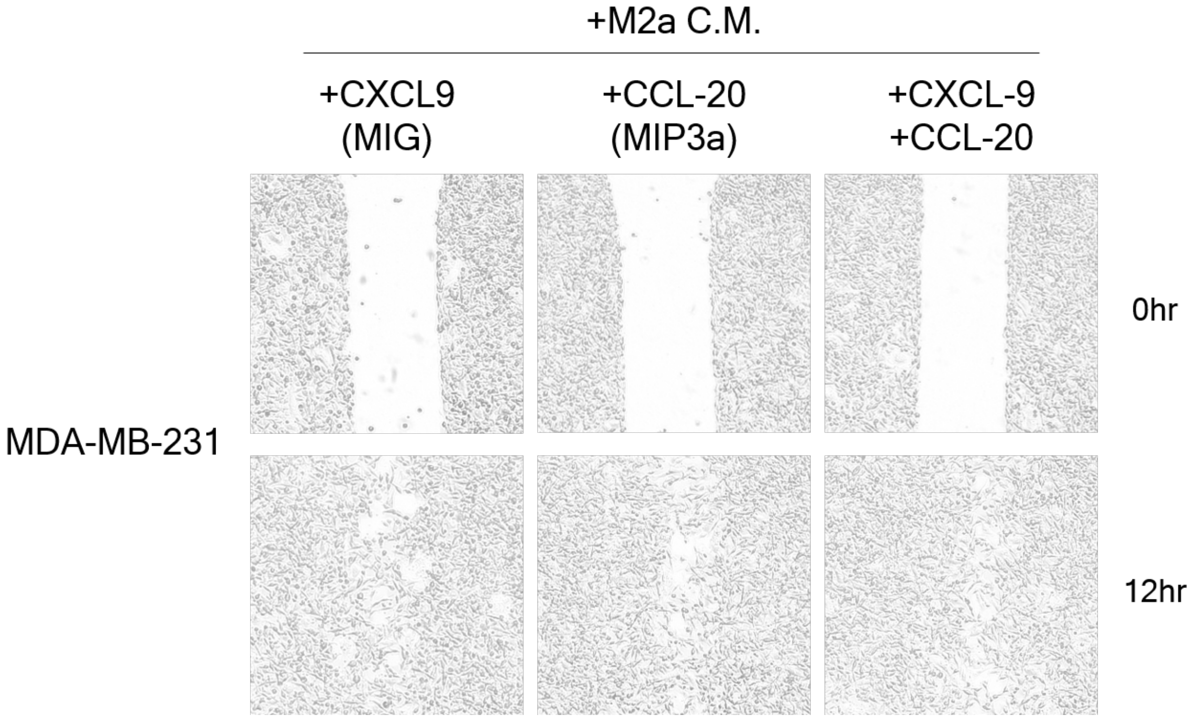

Supplemental Figure S4. Exogenous addition of inhibitory proteins, MIG, or MIP3a, do not affect M2a-induced migration. Results from MDA-MB-231 cells stimulated with M2a conditioned media, supplemented with recombinant CXCL-9 (MIG), CCL-20 (MIP3a), or both cytokines together, does not inhibit M2a macrophage induced migration. Images representative of 2 independent experiments.

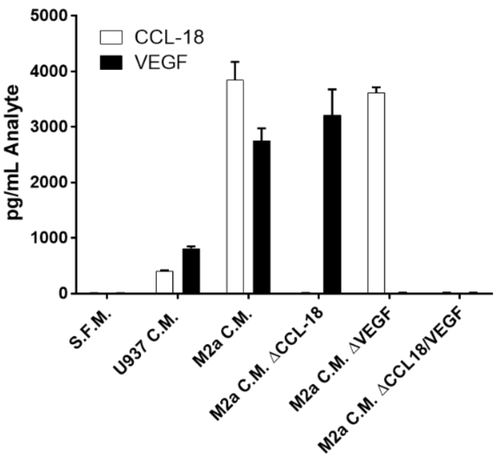

	CCL-18		VEGF	
	Avg. (pg/mL)	Std. Dev.	Avg. (pg/mL)	Std. Dev.
S.F.M.	3.65	1.60	1.24	2.63
U937 C.M.	408.21	10.54	813.34	36.98
M2a C.M.	3847.96	324.21	2755.21	219.31
M2a ΔCCL-18 C.M.	1.24	6.32	3219.88	456.32
M2a ΔVEGF C.M.	3621.23	94.25	10.63	6.32
M2a ΔCCL-18/ΔVEGF C.M.	11.65	6.32	16.32	2.36

Supplemental Figure 5. ELISA results from CCL-18 and/or VEGF analyte removal assay shows complete depletion of CCL-18 and/or VEGF in target concentrated medias. Raw values in pg/mL displayed right. Data is compiled from 3 independent experiments.

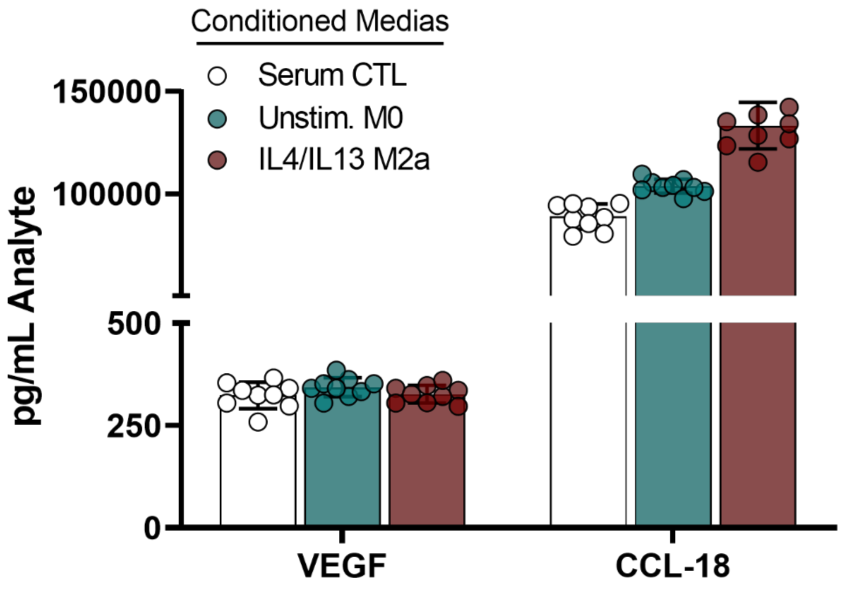

Supplemental Figure 6. ELISA results displaying levels of VEGF or CCL-18 in conditioned media from primary human macrophages. Results depict raw values of conditioned media from monocyte/macrophage serum-containing media (white), M-CSF polarized unstimulated macrophages (green), or M-CSF polarized IL-4/IL-13 stimulated M2a macrophages (red). Data is compiled from three independent experiments.

Gene	r^2	E %	Sequence (5'-3')
TLR4	0.981	101.832	F: TGCGTGAGACCAGAAAGC<br>R: TTAAAGCTCAGGTCCAGGTTC
S100A9	0.999	94.193	F: TGACAGAGTGCAAGACGATG<br>R: ACCAGCTCTTTGAATTCCCC
TLR2	0.998	94.709	F: AGACCTATAGTGACTCCCAGG<br>R: ACCCACACCATCCACAAAG
TGFβ	0.999	95.01	F: GCCTTTCCTGCTTCTCATGG<br>R: TCCTTGCGGAAGTCAATGTAC
DCR3	0.997	93.853	F: TCAATGTGCCAGGCTCTTC<br>R: CAGCCTCTTGATGGAGATGTC
CD86	0.985	104.115	F: TCCCTGATGTTACGAGCAATATG<br>R: ATCCAAGGAATGTGGTCTGG
MHC2	0.993	102.598	F: CAGAATGGAGACTGGACCTTC<br>R: TGTGCAGATTCAGACCGTG
CD68	0.981	100.29	F: CATCTCTGTACTGAACCCCAAC<br>R: CCATGTAGCTCAGGTAGACAAC
IL1α	0.982	114.731	F: TGTATGTGACTGCCCAAGATG<br>R: TTAGTGCCGTGAGTTTCCC
TGM2	0.988	123.303	F: TCAGCTACAATGGGATCTTGG<br>R: AAGGCAGTCACGGTATTTCTC
SOCS3	0.972	98.48	F: CTATGAGAAAGTCACCCAGCTG<br>R: CTTGTGCTTGTGCCATGTG
IL1β	0.943	108.846	F: ATGCACCTGTACGATCACTG<br>R: ACAAAGGACATGGAGAACACC
MGL1	0.958	107.126	F: CTTCGATCCCTACAGTGACG<br>R: CACATCACTAAGCTCCAGTACC
TNFα	0.975	117.833	F: GAGAAGCAACTACAGACCCC<br>R: CATGCTTTCAGTGCTCATGG
TLR1	0.99	111.135	F:<br>TTAGAAGAAATCAGGATAACAAAG<br>GC<br>R: GGCAAAATGGAAGATGCTAGTC
IL10	0.99	127.98	F: ATGCCCCAAGCTGAGAAC<br>R: GCCTTGCTCTTGTTTTCACAG
TLR8	0.973	125.292	F: ACAACCCGAAGGCAGAAG<br>R: GGCGCGAAATCATGACTTAAC
CLEC7a	0.996	106.401	F: TCGACTCTCAAAGCAATACCAG<br>R: CCACAGCTATCACCAGTATTACC
IL1R2	0.99	101.341	F: CGTGACAAAACTGACGTGAAG<br>R: CAGGACACAGCGGTAATAGC
IL1R1	0.987	91.55	F: AGATGACAGCAAGACACCTG<br>R:<br>TCTGAGGCAGTAAGATGAATTTCT
CD163	0.963	77.956	F: GAGTCCCTTCACCATTACTGTG<br>R: GACTTTCACTTCCACTCTCCC
CD80	0.952	71.178	F: AAGTGGAGTCTTACCCTGAAATC<br>R: AAGACCCTCCAGTGATGTTTAC
MRC1	0.972	127.571	F: ACAGTTATGCCTACCATGCC<br>R: CCCTCCAAAGCCTATACAAGC

*E % = Percent Efficiency

Supplemental Table 1. RT-qPCR primer sequences and corresponding efficiency scores utilized for this study.
